# Leuconychie transversale induite par la manucurie: y a-t-il un apport de la dermoscopie?

**DOI:** 10.11604/pamj.2014.18.39.3761

**Published:** 2014-05-10

**Authors:** Salim Gallouj, Fatima Zahra Mernissi

**Affiliations:** 1 Service de Dermatologie, Faculté de Médecine, CHU Hassan II, Fès, Maroc

**Keywords:** Leuconychie, manucurie, dermoscopie, ongle, leukonychia, manicure, dermoscopy, nail

## Abstract

La leuconychie transversale induite par la manucurie est une leuconychie secondaire au microtraumatisme transmis à la matrice unguéale. Nous rapportant une observation où la dermoscopie avait un intérêt considérable pour l'orientation diagnostic.

## Introduction

La leuconychie transversale induite par la manucurie est une leuconychie secondaire au microtraumatisme transmis à la matrice unguéale, souvent méconnue ou bien considérée carentielle. Nous rapportant une observation où la dermoscopie avait un intérêt considérable pour l'orientation diagnostic.

## Patient et observation

**Figure 1:**
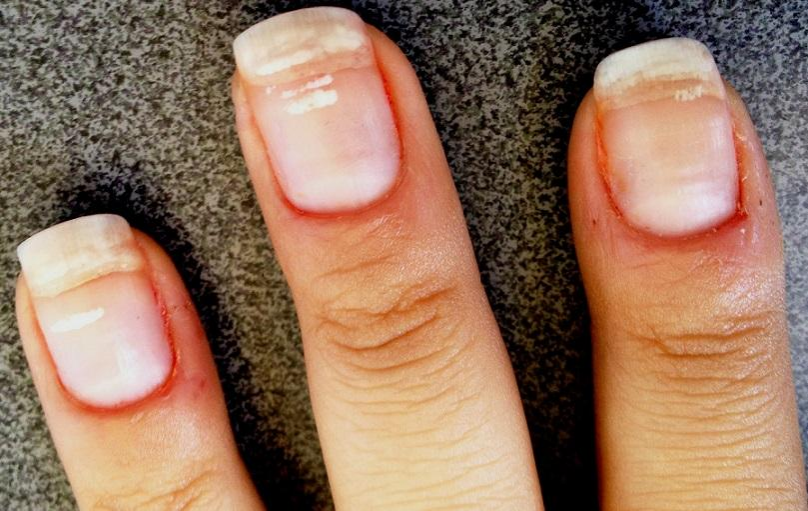
L'examen clinique montrait une leuconychie transversale diffuse à la majorité des ongles des mains avec une légère paronychie

**Figure 2:**
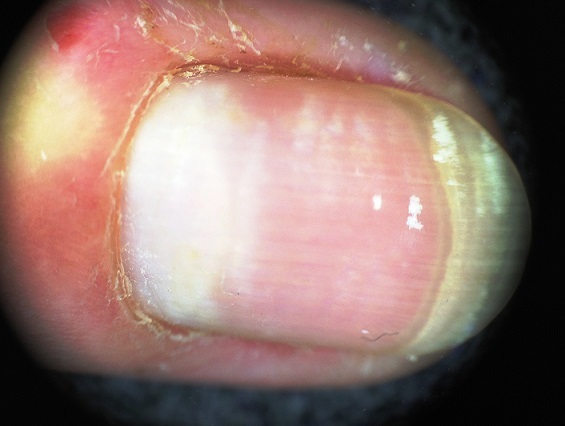
L'examen dermoscopique montre le refoulement de la cuticule, la présence de la paronychie, la macrolunule, les envies, d'une leuconychie transversale associée à une onycholyse.

**Figure 3:**
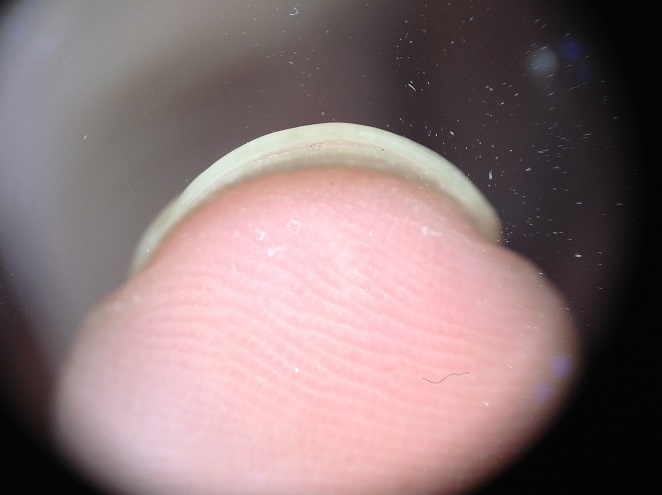
L'examen dermoscopique du bord libre montre une onycholyse

Patiente de 22 ans, ayant d'antécédent de manucurie qui présente depuis 1 an une coloration blanchâtre des ongles des mains évoluant dans un contexte d'apyrexie et de conservation de l’état général. La patiente ne rapporte pas la notion de contact avec les détergents, ni d'exposition arsenicale ni de prise de chimiothérapie. L'examen clinique (Figure 1) montrait une leuconychie transversale diffuse à la majorité des ongles des mains avec une légère paronychie. L'examen dermoscopique (Figure 2, Figure 3) montrait le refoulement de la cuticule, la présence de la paronychie chronique, les envies, la macrolunule et d'une leuconychie transversale associée à une onycholyse avec la ligne de détachement de la tablette et du lit unguéale est régulière évoquant une onycholyse post traumatique. Le reste de l'examen somatique était sans particularité. La numération formule sanguine, le bilan thyroidien, l'albumine, la bilan phosphocalcique étaient normaux. La sérologie VIH était négative. Le diagnostic d'une leuconychie transversale induite par la manucurie a été posé.

## Discussion

La Leuconychie est l′anomalie chromatique la plus courante de l′ongle et a été initialement classé par Unna en 1896 en leuconychie ponctuée,la Leuconychie striata et leuconychie totale. Un quatrième type, c'est la Leuconychie partielle, a été ajouté par Parkes Weber en 1918, Puis la leuconychie a été divisée en leuconychie vraie (participation de la tablette de l′ongle), et pseudoleuconychie (participation du lit de l′ongle) et la leuconychie apparente (onycholyse).la leuconychie vraie est en outre séparés en formes totale,sub-totale ou partielle, cette dernière peut être soit diffuse, transversale (strié), distale ou ponctuée [1].

La leuconychie transversale, aussi connue sous le nom des lignes de Mees, a été décrite pour la première fois par Mees en 1919, associé à une intoxication a l'arsenic. Bien que ces lignes sont classiquement associées à l'intoxication arsenicale, elles peuvent également être secondaire à diverses conditions comme les traumatismes c'est l'exemple de notre cas, les carences nutritionnelles, aux maladies systémiques, les maladies infectieuses et les médicaments notamment la chimiothérapie [2], également décrite secondaire à une hypocalcémie sévère [3].

Le diagnostic différentiel inclut la leuconychie transversale congénitale, et les pseudoleuconychie. La Pseudoleuconychie est principalement causée par des modifications oedémateuses des tissus du lit de l′ongle, en particulier en association avec hypoalbuminémie (ongle de Terry et les lignes de Muehrcke) ou bien à une insuffisance rénale [4].

La présence de leuconychie transversale secondaire à traumatisme est bien signalée. Toutefois, le délai entre la blessure et le développement de leuconychie est souvent oublié ou sont au mieux vague et rarement enregistré [5]. Les taches blanches et des stries transversales ont été expliqués par les microtraumatismes répétés que subit la matrice des ongles. La couleur blanche est causée par une anomalie structurale de la tablette qui décroît la transmission de la lumière. D'autres auteurs ont rapporté une anomalie de la kératinisation au niveau des bandes blanches. Les biopsies des leuconychie ont présumé être causé par les microtraumatismes. Elles ont noté une anomalie parakératosique dans le tiers inférieur de la tablette de l′ongle qui était fortement éosinophiles dans certains foyers près de la kératine sous-unguéale. Les onychocytes touchés diffèrent de celles de la plaque de l′ongle normalement kératinisé, dans lequel elles étaient plus grandes et de forme sphérique. Une désorganisation et une vacuolisation périnucléaire des onychocytes pourrait être noté par l′hématoxyline-éosine et d′acide périodique Schiff [6, 7].

Nous ne croyons que les microtraumatismes provoqués par les chocs répétés du bord libre de l'ongle conduit à endommager les onychocytes dans la matrice unguéale [6]. La dermoscopie est rarement décrite, elle montre une ou plusieurs bandes blanches transversales au fond de la plaque, avec une tablette unguéale normalement lisse en surface [8]. Chez notre patiente la dermoscopie avait un apport considérable pour l'orientation étiologique vers la manucurie en objectivant le refoulement de la cuticule, la présence de la paronychie chronique, les envies, la macrolunule avec des bandes leuconychique transversale associée surtout à une onycholyse traumatique confirmée par la régularité de la ligne de détachement de la tablette et du lit unguéale [8].

## Conclusion

La leuconychie transversale est une anomalie d’étiologies multiples dont l'origine traumatique reste sous estimée notamment secondaire à une manucurie. La dermoscopie a fourni des éléments d'orientation notamment l'onycholyse traumatique.

## References

[1] Chaudhry SI, Black MM (2006). True transverse leuconychia with spontaneous resolution during pregnancy. Br J Dermatol..

[2] Ceyhan AM, Yildirim M, Bircan HA, Karayigit DZ (2010). Transverse leukonychia (Mees’ lines) associated with docetaxel. J Dermatol..

[3] Foti C, Cassano N, Palmieri VO, Portincasa P, Conserva A, Lamuraglia M, Palasciano G, Vena GA (2004). Transverse leukonychia in severe hypocalcemia. Eur J Dermatol..

[4] Fujita Y, Sato-Matsumura KC, Doi I, Takaoka K (2007). Transverse leukonychia (Mees’ lines) associated with pleural empyema. Clin Exp Dermatol..

[5] Bowling JC, McIntosh S, Agnew KL (2004). Transverse leukonychia of the fingernail following proximal nail fold trauma. Clin Exp Dermatol..

[6] Maino KL, Stashower ME (2004). Traumatic transverse leukonychia. Skinmed..

[7] Baran R, Perrin C (1995). Transverse leukonychia of toenails due to repeated microtrauma. Br J Dermatol..

[8] Piraccini BM, Bruni F, Starace M (2012). Dermoscopy of non-skin cancer nail disorders. Dermatol Ther..

